# Supplementary material for: Spin orbit torques and Dzyaloshinskii-Moriya interaction in dual-interfaced Co-Ni multilayers
Source: Sci Rep. 2016 Sep 7;6:32629. doi: 10.1038/srep32629 (PMC5013523; doi:10.1038/srep32629)
Supplement: Supplementary Information [file srep32629-s1.pdf]

Supplementary Information

Spin orbit torques and Dzyaloshinskii-Moriya interaction in dual-interfaced Co-Ni multilayers

Jiawei Yu[1], Xuepeng Qiu[1], Yang Wu[1], Jungbum Yoon[1], Praveen Deorani[1], Jean Mourad Besbas[1], Aurelien Manchon[2], and Hyunsoo Yang[1*]

[1] Department of Electrical Engineering and Computer Engineering, National University of Singapore, 117576, Singapore

[2] Division of Physical Science and Engineering, King Abdullah University of Science and Technology (KAUST), Thuwal 23955, Saudi Arabia

1. Second harmonic measurements to quantify spin orbit effective fields

The spin orbit effective fields are quantified by using the second harmonic measurement technique[S1,2]. The measurement schematics are presented in Fig. S1(a,b) for the longitudinal and transverse measurements, respectively. Blue arrows represent the magnetization directions, gray arrows show the applied field (H) directions and orange arrows give the ac charge current (I_{ac}) directions. A sinusoidal current (I_{ac}) with the magnitude of 10 mA and the frequency of 13.7 Hz is applied to the devices and two lock-in amplifiers are used to measure the 1st and 2nd harmonic voltages across the Hall bar. The measurement results are shown in Fig. S1(c) and S1(d) for the longitudinal and transverse measurements, respectively. One should note that the longitudinal SOT effective field (H_L) and transverse SOT effective field (H_T) are coupled, and both contribute to the 2nd harmonic signal in any measurement geometry. We use the data from the Ta capped device as an example. The black line in Fig. S1(c) shows the 1st harmonic voltage and the red line gives the result of 2nd harmonic voltage. In the longitudinal measurement data [Fig. S1(c)], a positive peak of the 2nd harmonic voltage is observed in the positive field region and a negative peak exists in the negative field region. In the transverse measurement data [Fig. S1(d)], there is a positive peak in the positive and negative field regions. The 2nd harmonic voltage is then fitted by a model described in Ref. S2 and the fitting results are shown in Fig. S1(e,f). The anisotropy field (H_k), longitudinal SOT effective field (H_L), and transverse SOT effective field (H_T) are obtained

from the fitting. The ratio of the anomalous Hall effect (AHE) and the planar Hall effect (PHE), which will be discussed in detail in the next section, is considered during the fitting.

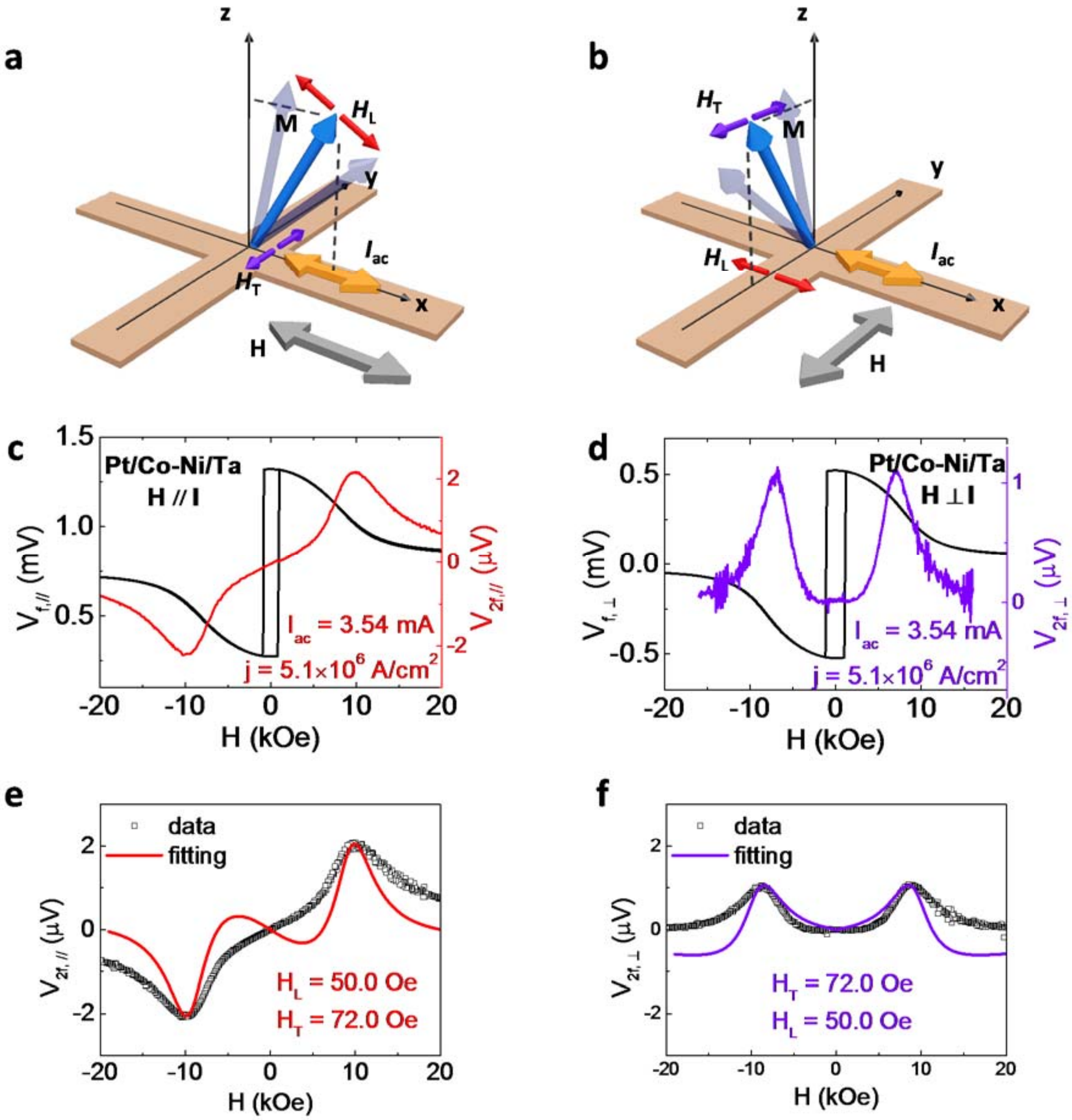

Fig. S1. Measurements of SOT effective fields. The longitudinal (a) and transverse (b) measurement schematics to extract H_L and H_T. The first and second harmonic voltages of longitudinal (c) and transverse (d) geometry. (e,f) the fitting of the second harmonic voltages. The obtained H_L and H_T values are indicated in the graphs.

The SOT effective fields are known to have complex angular (dependence on magnetization direction) features[S2-4]. For a quantitative comparison between different samples, we fit the SOT effective fields from the peak/dip values of the second harmonic signal. Even though this method leads to the less satisfactory fitting in the field region away from the peak/dip position, it is still capable of capturing the strength of SOT effective fields.

2. Measurements of the anomalous Hall resistance (R_{AHE}) and planar Hall resistance (R_{PHE})

The Hall resistance with considering both AHE and PHE can be written as

$$R = R_{AHE} \sin\theta + R_{PHE} \cos^2\theta \sin 2\phi ,$$

where the first term is the AHE contribution and the second term gives the PHE contribution. The angle θ and ϕ are defined in Fig. S2(a). The R_{AHE} is obtained by measuring the out-of-plane loop of the Hall resistance. Figure S2(b) shows the AHE measurement result in a Ta capped device and the $2R_{AHE}$ value is indicated in the graph. Because of PHE, the Hall resistance changes with the in-plane angle between the current and the magnetization direction (θ_{IM}) as shown in Fig. S2(c). Thus the planar Hall resistance, R_{PHE}, is obtained by measuring the in-plane angular dependence of the Hall resistance. A strong in-plane magnetic field (6 T) is applied to the device to saturate the magnetization in plane. The Hall resistance as a function of θ_{IM} is measured. Figure S2(d) shows the result of R_H as a function of θ_{IM}, and the $2R_{PHE}$ value can be extracted as the difference of the Hall resistance between its maximum and minimum values (as indicated in the graph). The ratio of R_{PHE} and R_{AHE} is then calculated and used as a correction parameter to extract the SOT effective field values. The extracted R_{PHE}/R_{AHE} values are 0.481, 0.557, 0.326 and 0.598 for MgO, Cu, Pt and Ta capped devices, respectively. The R_{PHE}/R_{AHE} values are large in our devices and thus the effect of R_{PHE} cannot be ignored.

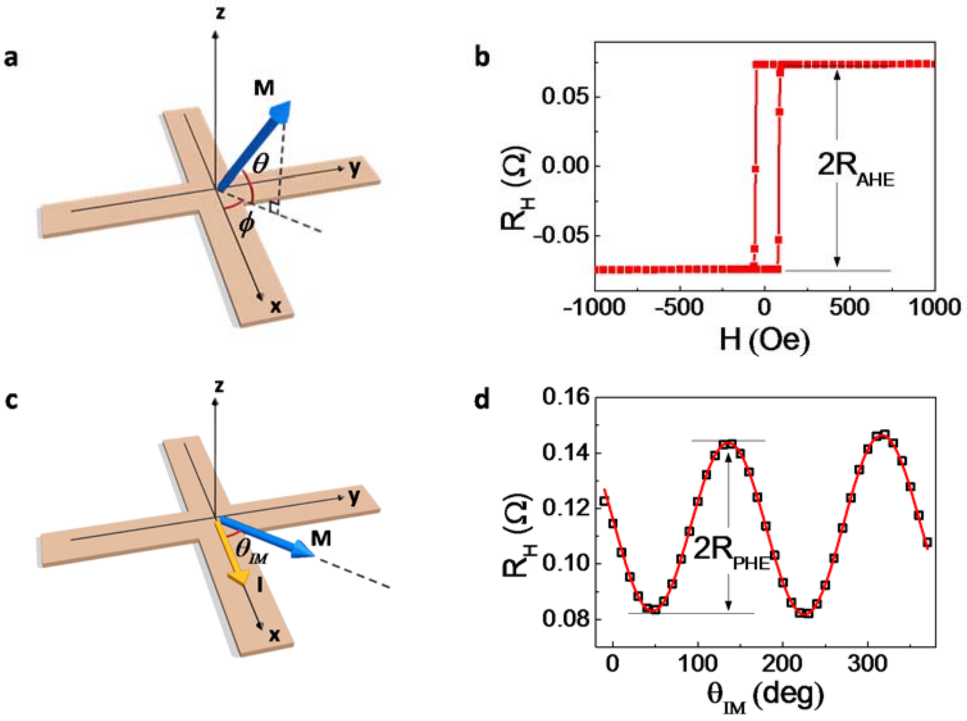

Fig. S2. Measurement schematics of R_{AHE} and R_{PHE}. (a) Definition of the angles used in the Hall resistance equation. The blue arrow shows the magnetization (M). (b) The loop of the Hall resistance as a function of applied out-of-plane magnetic field. (c) The definition of the angle (θ_{IM}) between the current and magnetization. The orange arrow shows the direction of the applied current (I). (d) The measurement result of the R_{PHE}.

3. SOT current induced magnetization switching

The SOT current induced magnetization switching with a longitudinal assist field (H_{assist}) along or opposite to the current direction is studied. As presented in Fig. S3, the current direction is either $+\hat{x}$ or $-\hat{x}$ ($\vec{J} = J\hat{x}$).

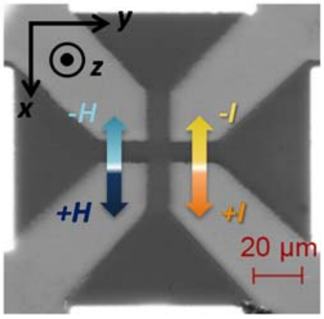

Fig. S3. Measurement schematic of SOT current induced magnetization switching.

The H_{assist} is swept from 0 to 1 T in both $+\hat{x}$ and $-\hat{x}$ directions, and the SOT induced switching curves are measured. The switching direction is determined by the sign of $\vec{J} \cdot \vec{H}_{assist}$. A counterclockwise switching is shown in red in the phase diagrams [Fig. S(4)], and a clockwise switching is shown in blue in the phase diagrams. As can be seen in Fig. S4(a-d), normal switching behaviors are observed in MgO and Pt devices, showing that full switching can happen with a small H_{assist} (~ 50 – 200 Oe). The threshold switching current (I_{sw}) gradually becomes smaller as the H_{assist} increases. However, the Ta capped device behaves differently in the low field region (-1000 Oe – 1000 Oe), showing incomplete switching [Fig. S4(e-f)]. The incomplete current induced switching behavior in the Ta capped device has been discussed in the main manuscript. The normal full switching can only happen, when the DMI induced chiral spin texture is eliminated by a large in-plane assist field.

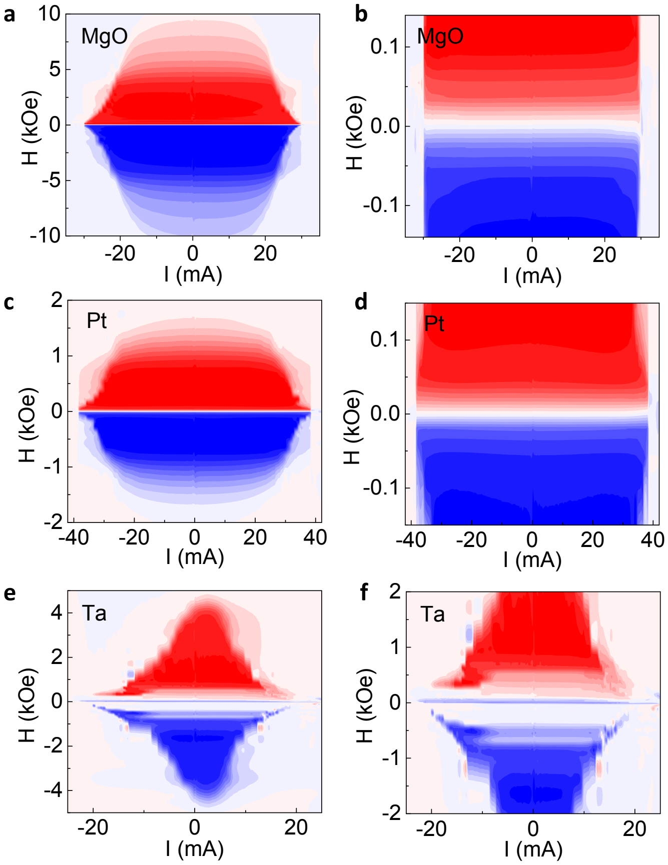

Fig. S4. SOT current induced magnetization switching of the MgO, Pt and Ta capped devices with different assist fields. (a) The phase diagram of the MgO capped device. The red and blue colors represent counterclockwise and clockwise switching behaviors, respectively. (b) Magnification of a in the low field region. (c,d) The phase diagram of the Pt capped device. (e,f) The phase diagram of the Ta capped device.

4. Polar Kerr images of SOT current induced magnetization switching process

The polar Kerr microscope is utilized to monitor the SOT current induced switching process simultaneously with the electrical measurement of the switching curves. The Kerr measurement schematic is shown in Fig. S3. The switching processes in MgO, Cu, Pt and Ta devices with a 400 Oe assist field are measured. As stated in the main manuscript, a large assist field up to 1000 Oe, is needed to obtain full switching in the Ta capped device. Thus we also monitor the switching process in the Ta capped device with a 1000 Oe assist field. The switching process in the Cu capped device with a 400 Oe assist field as well as the switching behaviors in the Ta capped device with a 400 and 1000 Oe assist field are presented in the main manuscript (Fig. 2). The normal switching process in MgO and Pt capped devices with a 400 Oe assist field are shown in Fig. S5(a,b) and S5(c,d), respectively. Similarly to Cu capped device, full switching is obtained in MgO and Pt capped devices with a small assist field of 400 Oe.

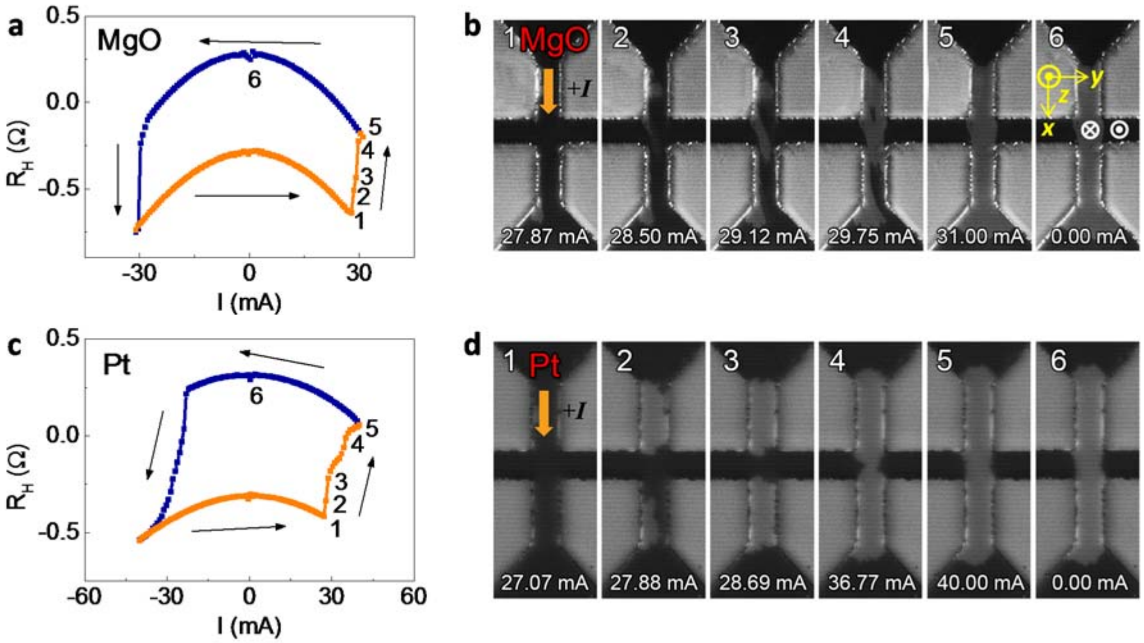

Fig. S5. Measurements of SOT current induced switching. (a) The SOT current induced switching curve in MgO capped device with a 400 Oe assist field. (b) The DW configurations captured by Kerr microscope simultaneously with the SOT current induced switching measurement. The sample is pre-magnetized pointing out of plane (dark part in the Kerr image) and the light parts show the switched region (the magnetization pointing into the plane). The magnitude of applied current is indicated at the bottom. The switching curve and Kerr images of the Pt capped device are displayed in (c) and (d), respectively.

5. Vibrating sample magnetometer measurements

The saturation magnetization (M_S) and the anisotropy field (H_k) are obtained by vibrating sample magnetometer (VSM) along both easy and hard axis of the as-deposited films as shown in Fig. S6. The easy and hard axis curves merge when the applied field reaches H_k (marked in the first figure).

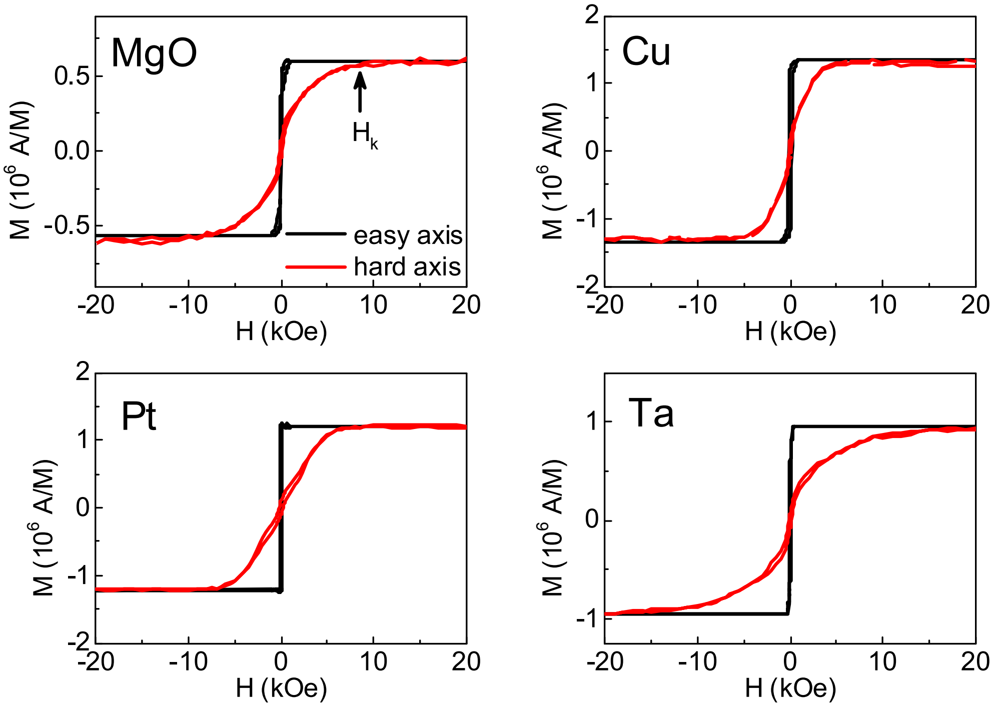

Fig. S6. VSM measurement results. The red curve shows the loop obtained along the hard axis (in-plane) of the film. The black curve presents the loop obtained along the easy axis (out-of-plane) of the film.

6. Phenomenological modeling of chiral domain wall dynamics

In order to understand the abnormal current-driven switching properties of the Ta capped samples discussed in the main text, we consider the model that accounts for a general tilting of the domain wall[S3] as illustrated in Fig. S7. In this frame, the magnetization is $\vec{m} = (\sin\theta\sin\psi, -\sin\theta\cos\psi, \cos\theta)$ and $\theta = 2\tan^{-1}\left\lfloor e^{s(x\cos\chi + y\sin\chi - q\cos\chi)/\Delta}\right\rfloor$, where Δ is the DW width. s = 1 corresponds to a domain wall from down ($x < q$) to up ($x > q$), while s = -1 corresponds to a domain wall from down ($x < q$) to up ($x > q$). The magnetic domain wall is

affected by spin-orbit fields $H_T\vec{y}, H_L\vec{y}\times\vec{m}$, spin transfer torques (STT) $b_J\left(-\partial_x\vec{m}+\beta\vec{m}\times\partial_x\vec{m}\right)$ and an applied magnetic field $H_x\vec{x}+H_y\vec{y}$. In the STT expression, b_J correspond to the efficiency of the adiabatic STT, and β is the ratio of non-adiabatic and adiabatic STT. The Landau-Lifshitz-Gilbert equation then can be written as

$$\left(\partial_t+b_J\partial_x\right)\vec{m}=-\gamma\vec{m}\times\left(\vec{H}_{eff}+H_T\vec{y}\right)+\vec{m}\times\left[\alpha\partial_t\vec{m}-\gamma H_L\left(\vec{y}\times\vec{m}\right)+\beta b_J\partial_x\vec{m}\right]. \qquad (1)$$

In the above equation, α is the Gilbert damping parameter and γ is the gyromagnetic ratio. The effective field is defined $\vec{H}_{eff}=-\delta W/\delta\vec{m}$ and the magnetic energy density is

$$W=A\sum_i\left(\partial_i\vec{m}\right)^2-K_\perp m_z^2-D\vec{m}\cdot\left(\left(\vec{z}\times\vec{\nabla}\right)\times\vec{m}\right)-K_D\left(\vec{m}\cdot\vec{u}\right)^2-M_s\vec{m}\cdot\vec{H}+V\left(\vec{m}\right). \qquad (2)$$

The first term is the magnetic exchange and A is the exchange stiffness constant, the second term is the effective perpendicular magnetic anisotropy and $K_\perp$ is the effective magnetic anisotropy constant, the third term represents Dzyaloshinskii-Moriya interaction and D is the DMI constant, the fourth term arises from the dipolar interaction that favors the Bloch configuration over the Néel configuration of the domain wall ($\vec{u}=\left(\sin\psi,-\cos\psi,0\right)$) and $K_D=N_x\mu_0M_S^2/2$ is the DW anisotropy energy density, and the fifth term is the applied magnetic field ($\vec{H}$) and M_S is the saturation magnetization. The last term represents the (possibly chiral) pinning potential arising from the specific geometry of the Hall bar as well as the edge roughness. Although we have no explicit information about the form of this term, its impact is essential to understand the dynamics of the chiral domain wall as discussed below.

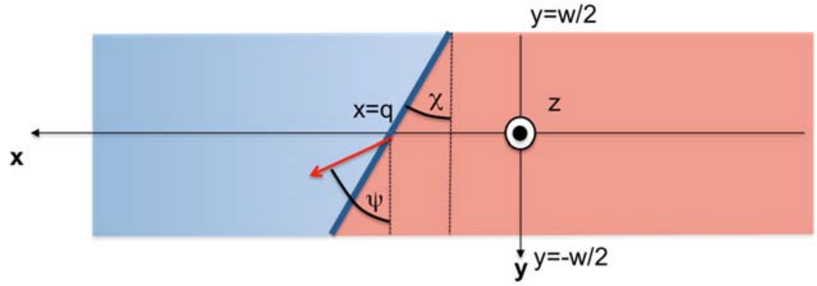

Fig. S7. Schematics of the tilted magnetic domain wall. The red and blue regions correspond to oppositely magnetized domains, and the magnetic wall, located as the position $x = q$, is tilted with an angle χ with respect to the transverse direction y.

This system can be solved following Lagrange-Raleigh formalism and provides three coupled dynamical equations for the domain wall position q, the azimuthal angle of the wall ψ and the tilting angle χ.[S5] The resulting equations are

$$\frac{\cos\chi}{\Delta}\partial_t q=\left(\frac{\cos\chi}{\Delta}(1+\alpha\beta)b_J-s\alpha\frac{\gamma\pi}{2}H_L\sin\psi\right)+\frac{\gamma\pi}{2}\left(H_{DMI}\cos(\psi-\chi)+s\frac{H_D}{2}\sin2(\psi-\chi)-sH_x\cos\psi-s(H_y+H_T)\sin\psi\right)$$

$$(1+\alpha^2)\partial_t\psi=s\frac{\cos\chi}{\Delta}(\beta-\alpha)b_J-\alpha\frac{\gamma\pi}{2}\left(sH_{DMI}\cos(\psi-\chi)+\frac{H_D}{2}\sin2(\psi-\chi)-H_x\cos\psi-\left(H_y+H_T-\frac{H_L}{\alpha}\right)\sin\psi\right)$$

$$\frac{\alpha\pi^2}{12}\left[\tan^2\chi+\frac{w^2}{\Delta^2\pi^2}\frac{1}{\cos^2\chi}\right]\partial_t\chi=s\frac{\gamma\pi}{2}\left(H_{DMI}\cos(\psi-\chi)+\frac{H_D}{2}\sin2(\psi-\chi)\right)-\gamma\frac{\sigma_{DW}}{2\Delta M_s}\tan\chi$$

where

$$\Delta=\sqrt{A/K_\perp},\ H_{DMI}=D/\Delta M_s,\ H_D=4K_D/\pi M_s$$

$$\sigma_{DW}=2\left(\frac{A}{\Delta}+K_\perp\Delta\right)-2\gamma\Delta K_D\cos^2(\psi-\chi)+\pi\Delta M_s\left(sH_{DMI}\sin(\psi-\chi)-H_x\sin\psi+(H_y+H_T)\cos\psi\right)+V(\vec{m}).$$

In the above equations, we assume that the pinning potential only influences the tilting of the domain wall, as seen experimentally, and hence we did not include it in the dynamical equations of the domain wall position and azimuthal angle. In steady state, the velocity is given by

$$\partial_t q=\frac{\beta}{\alpha}b_J-s\frac{\gamma\pi\Delta}{2\alpha}H_L\frac{\sin\psi}{\cos\chi}, \tag{3}$$

and is determined by the ratio $\sin\psi/\cos\chi$. The azimuthal angle ψ is determined by the equation

$$sH_{DMI}\cos(\psi-\chi)+\frac{H_D}{2}\sin2(\psi-\chi)-H_x\cos\psi-\left(H_y+H_T-\frac{H_L}{\alpha}\right)\sin\psi=s\frac{2\cos\chi}{\gamma\pi\Delta}\left(\frac{\beta}{\alpha}-1\right)b_J, \tag{4}$$

while the tilting angle fulfills

$$\gamma\frac{\sigma_{DW}}{2\Delta M_s}\tan\chi=s\frac{\gamma\pi}{2}\left(H_{DMI}\cos(\psi-\chi)+\frac{H_D}{2}\sin2(\psi-\chi)\right). \tag{5}$$

The exact form of σ_{DW} is difficult to determine explicitly since it depends on the pinning potential, which is unknown. In the narrow magnetic wires we consider in this work, edge roughness and asymmetric bubble nucleation are expected to dramatically influence the tilting angle of the domain wall. Therefore, in the following we do not attempt to calculate this tilting using Eq. (5). In contrast, once the tilting angle χ is known (from the MOKE images for instance), one can deduce the azimuthal angle ψ using Eq. (4). At this stage, it is also important

to notice that the adiabatic and non-adiabatic torques are negligible in the system considered. Indeed, due to the current shunting in the non-magnetic metals below and on top of the magnetic layer, it is expected that the domain wall velocity is dominated by the spin Hall effect and more specifically $\partial_t q \approx -s\frac{\gamma\pi\Delta}{2\alpha}H_L\frac{\sin\psi}{\cos\chi}$.

7. DMI and SOT switching in sample with changing capping Ta thickness

Extended experiments have been carried out to explore the SOT switching and DMI in thicker Ta capping devices. We have fabricated the sample with the following structures of Pt(4)/Co(0.1)/[Ni(0.1)/Co(0.1)]$_4$/Ta(X)/MgO(1)/SiO_2(3 nm), where X = 2, 3, 4, and 8 nm. The MgO/SiO_2 capping is used to prevent the Ta cap from being oxidized. The DMI and SOT current induced switching measurements have been carried out similarly as stated in the main text. The measurement results of X = 3 nm are shown in Fig. S8, for example.

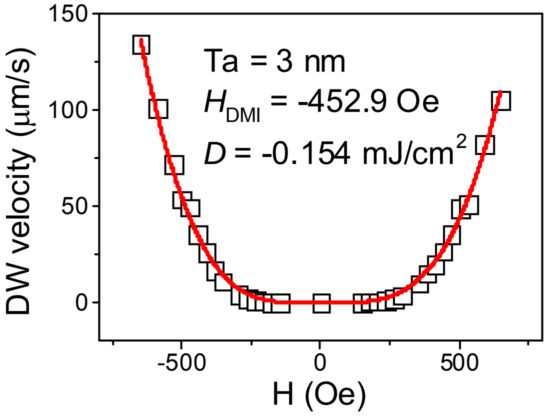

Fig. S8. The asymmetric domain wall creep velocity as a function of the applied in-plane magnetic field for X = 3 nm Ta capping films. The symbols are the experimental results and the solid line shows the fitting curve.

The SOT current induced switching measurements are also performed in the samples with different Ta capping thicknesses. As can be seen in Fig. S9(a), the anhysteretic current switching still exists (the data from Ta = 3 nm sample are shown as an example) when the assist field is not large enough to overcome the negative DMI field (H_{assist} = 400 Oe, red lines). Hysteretic switching curves are obtained when a large assist field is applied (H_{assist} = 1000 Oe, black lines). For the Ta = 8 nm sample as shown in Fig. S9(b), H_{assist} = 400 Oe is large enough to overcome the H_{DMI} field (-274.2 Oe), therefore the current induced switching shows hysteresis loops.

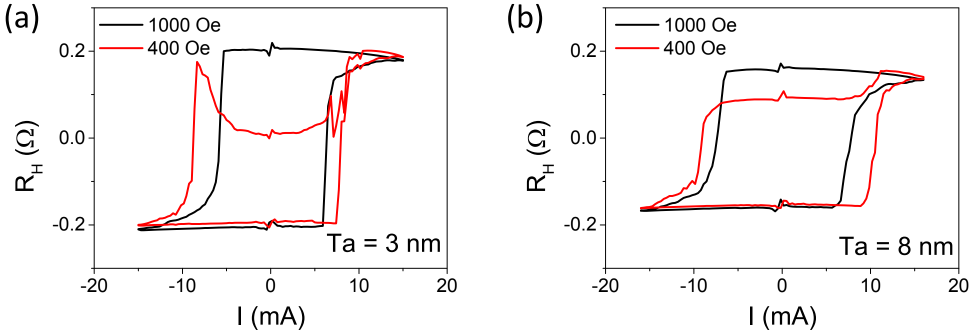

Fig. S9. The SOT induced magnetization switching loops with a 400 Oe assist field (red lines) and 1000 Oe assist field (black lines) in (a) Ta = 3 nm sample, and (b) Ta = 8 nm sample.

We also explore the minimum assist field that is required for a hysteretic switching ($H_{\text{assist, min}}$) with different Ta capping thicknesses. VSM measurements have been carried out for all the films to extract the M_S values for the DMI calculation. All the measurement results ($H_{\text{assist, min}}$, H_{DMI}, D and M_S) are summarized in Table S1.

TABLE S1. Summary of experimental results with different Ta capping thicknesses.

**Capping**	**Ta 2nm**	**Ta 3nm**	**Ta 4nm**	**Ta 8nm**	**Ta 4nm/Ru 4nm**
$H_{\text{assist, min}}$ **(Oe)**	1000	600	> 400	500	300
H_{DMI} **(Oe)**	-1038.6	-452.9	*	-274.2	*
D **(mJ/cm^2)**	-0.394	-0.15437	*	-0.10546	*
M_S **(emu/cc)**	749.47	726.12	708.82	924.55	638.40

*Difficult to extract the DMI value because of the small domain size in the film.

The minimum assist field and the DMI field with respect to the capping Ta thickness are plotted in Fig. S10. H_{DMI} and $H_{\text{assist, min}}$ are clearly correlated and comparable to each other in all Ta capped samples (regardless of the Ta thickness). However, the HM layer thickness dependence of DMI is still not clear[S6], and more works need to be done in the future.

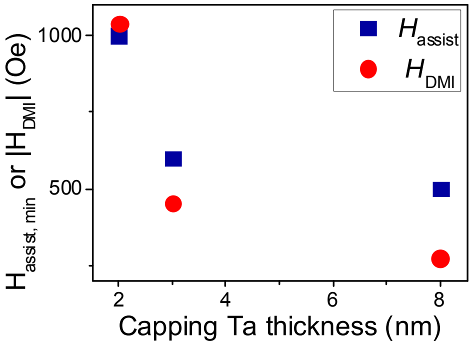

Fig. S10. The $H_{\text{assist, min}}$ and H_{DMI} as a function of capping Ta thickness.

8. Current shunting effect in Pt/Co-Ni/Cap tri layer systems

In the Pt/Co-Ni/Capping tri layer system, the current densities in the bottom Pt and top capping layer are different due to the different resistivity of different layers. In this section, we discuss the SOT magnitudes in different layers by considering the current shunting effect.

The resistivity of Ta, Pt, Cu, and Co-Ni used in our work is measured to be 156.44 μΩ·cm, 35 μΩ·cm, 20.26 μΩ·cm, and 28.55 μΩ·cm, respectively. We utilize the 1D current shunting model, where the stack is regarded as a parallel resistor network. The current density in the bottom Pt (HM) and capping (cap) layers are calculated by, respectively,

$$J_{HM} = \frac{1}{\rho_{HM}} \frac{t_{FM} + t_{HM} + t_{Cap}}{t_{FM}/\rho_{FM} + t_{HM}/\rho_{HM} + t_{Cap}/\rho_{Cap}} J_S , \tag{6}$$

$$J_{Cap} = \frac{1}{\rho_{Cap}} \frac{t_{FM} + t_{HM} + t_{Cap}}{t_{FM}/\rho_{FM} + t_{HM}/\rho_{HM} + t_{Cap}/\rho_{Cap}} J_S , \tag{7}$$

where J_S is the total current density in the tri layer system. ρ_{HM}, ρ_{FM}, and ρ_{cap} are the resistivity of the bottom Pt, Co-Ni, and capping layers, respectively. t_{HM}, t_{FM}, and t_{cap} stand for the each layer thickness.

To compare the SOT contributions from different layers, we first list the raw SOT effective fields data without normalization to the total current density ($H_{L/T}$) in the first two columns in Table S2. The current magnitudes in the upper and lower HMs are summarized in the last two columns.

TABLE S2. Summary of raw SOT effective fields (without normalization to the current density) and current densities in different layers.

**Structure**	**H_L (Oe)**	**H_T (Oe)**	**$J_{Pt, bottom}$ (10^6 A/cm^2)**	**$J_{Capping}$ (10^6 A/cm^2)**
Pt/Co-Ni/Pt	5.4	3.09	9.95	4.98
Pt/Co-Ni/Ta	50	72	12.74	1.43
Pt/Co-Ni/Cu	9.72	8.75	8.26	7.14
Pt/Co-Ni/MgO	15.6	13	13.86	0

We then calculate SOT effective fields normalized with the current density of the bottom Pt layer in Pt/Co-Ni/Cu and Pt/Co-Ni/MgO structures. The H_L/J_{Pt} and H_T/J_{Pt} from bottom Pt in these two structures, which are single HM systems, are summarized in Table S3. The normalized effective fields ($H_{L/T}/J_{Pt}$) in Cu and MgO capped structures are similar, confirming that Cu and MgO does not contribute to SOT. The $H_{L/T}$ contributed from bottom Pt in Pt/Co-Ni/Pt and Pt/Co-Ni/Ta structures (double HM systems) are then calculated by

$$H_{L/T} = H_{L/T,0}(\frac{J_{Pt}}{J_{Pt,0}}), \tag{8}$$

where $H_{L/T,0}$ and $J_{Pt,0}$ are the SOT effective fields and current density in the Pt layer in Pt/Co-Ni/MgO structure, respectively. J_{Pt} is the current density in the bottom Pt layer in Pt/Co-Ni/Pt or Pt/Co-Ni/Ta structures. Here, we use $H_{L/T}$ in a single HM system as a reference to extract the bottom Pt contribution in double HM systems. The $H_{L/T}$ from the capping HM are calculated by subtracting the bottom Pt contribution from the total $H_{L/T}$. The SOT effective fields normalized by the current density in each layer are calculated by $H_{L/T}/J$, where J is the current density in that HM layer. The $H_{L/T}/J$ results are shown in Table II in the main text.

TABLE S3. SOT effective fields normalized to the current density in single HM systems.

**Structure**	**H_L/J from bottom Pt (Oe per 10^8 A/cm^2)**	**H_T/J from bottom Pt (Oe per 10^8 A/cm^2)**
Pt/Co-Ni/Cu	235.29	211.81
Pt/Co-Ni/MgO	225.18	187.65

As shown in Table II, the contributions of $H_{L/T}/J$ from the capping layers vary substantially. The Cu and MgO capping layer contributes almost zero SOT, which agrees well with the theoretical prediction. The Pt capping layer contributes to small positive SOT, causing a cancellation effect of the total SOT in the tri layer system. The Ta capping layer contributes to a large negative SOT and gives rise to a much larger total SOT as compared to the other structures. The Ta contribution with considering the current shunting effect is larger than that without considering the current shunting effect.

9. OOMMF simulations of SOT and pinning effects on DW tilting

As it is recently reported[S5,7], the DW tilting is affected by various factors such as STT, SOT, applied magnetic field, and DMI. In order to understand the tilting angle change in Fig. 5(d), OOMMF simulations are carried out on submicron sized wires with the dimension of 100 nm width and 400 nm length as indicated in Fig. S11. To simulate the DW configuration in a wire with injecting currents and applied longitudinal fields, we consider the Landau–Lifshitz–Gilbert (LLG) equation including SOT terms,

$$\frac{d\mathbf{m}}{dt} = -|\gamma|\mathbf{m}\times\mathbf{H}_{\text{eff}} + \alpha\left(\mathbf{m}\times\frac{d\mathbf{m}}{dt}\right) + |\gamma|\beta\varepsilon\left(\mathbf{m}\times\mathbf{m}_p\times\mathbf{m}\right) - |\gamma|\beta\varepsilon'\mathbf{m}\times\mathbf{m}_p, \tag{9}$$

where $\mathbf{m} = \dfrac{\mathbf{M}}{M_S}$ is the reduced magnetization, γ is the gyromagnetic ratio, $\beta = \left|\dfrac{\hbar}{\mu_0 e}\right|\dfrac{J}{tM_S}$, $\mathbf{m}_p$ is the electron polarization direction, $\varepsilon = \dfrac{P\Lambda^2}{\left(\Lambda^2+1\right)+\left(\Lambda^2-1\right)\left(\mathbf{m}\cdot\mathbf{m}_p\right)}$, and ε' is the secondary spin transfer term. The cell size of 2 × 2 × 0.4 nm^3, M_S = 0.87 × 10^6 A/m, K_U = 0.8 × 10^6 J/m^3, an exchange stiffness constant of 1.0 × 10^{11} J/m, and damping constant of 0.1 are used. Electrical current pulses with a rising time of 65 ps are applied to the wire as indicated by the orange arrow in Fig. S11(a). An assist bias field (H_x) of 200 mT is applied along the current direction as presented by the blue arrow. We use positive SOT (the same sign as the Pt spin Hall angle) and DMI = -1.0 mJ/m^2, which is the case with our Pt/Co-Ni/Ta structure in Fig. 5(d).

Figures S11(a-g) show the transient snapshots at 660 ps. The current density is indicated in the left corner of each figure. DW tilting is observed in Fig. S11(a-f), and the tilting angle (indicated in the right corner of each figure) reduces as the injected current density increases. This

simulation results agree with our experiment data in Fig. 5(d), in which a larger current, thus larger SOT, tends to cause a small tilting angle.

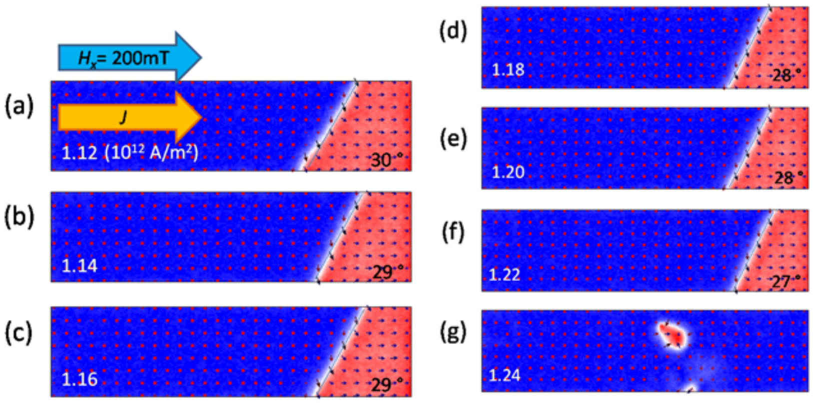

Fig. S11. OOMMF simulation results of DW tilting in submicron sized wires with different current densities.

Furthermore, we have studied the pinning effect on the DW tilting. We add a notch with the size of 16 × 16 nm^2 in the middle of the wire indicated as a white square in Fig. S12. An electrical current pulse with a density of 1.4 × 10^{12} A/m^2 and rising time of 65 ps is applied to the wire. Snapshots of the DW shape at different times are shown in Fig. S12(a-e). As can be seen, the tilting angle becomes smaller partially as it passes the pinning site (see Fig. S12(e)), showing the effect of a pinning site on the DW tilting. This result may account for the experimental observation of a smaller tilting angle in panel 4 of Fig. 5(d) due to a pining site when a DW exits the wire. In summary, we show that DW tiling can be affected by the current density and pinning site, which may explain the different DW tilting angles in Fig. 5(d).

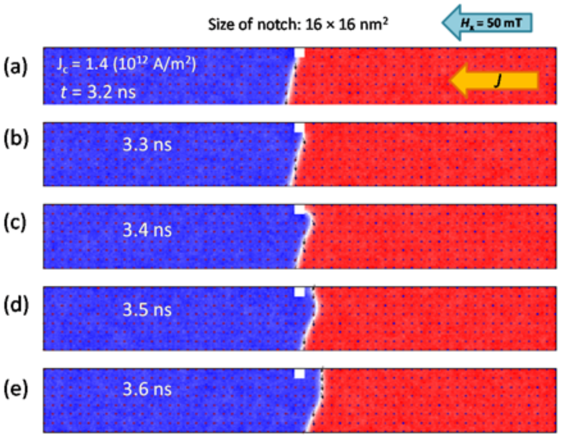

Fig. S12. Simulation results of pinning effect on the DW tilting. (a-e) the snapshots of the DW shape at different times.

10. Angular dependence of SOT effective fields

As can be seen in Fig. S13, there is an abnormal negative second harmonic voltage in the low field region. Our data look abnormal compared to the conventional H_T data.[S3] This is mainly attributed to the large planar Hall effect (PHE) in our Co/Ni devices. It should be noted that the Nernst-Ettingshausen effect is corrected in our data using the method described in Ref. S3.

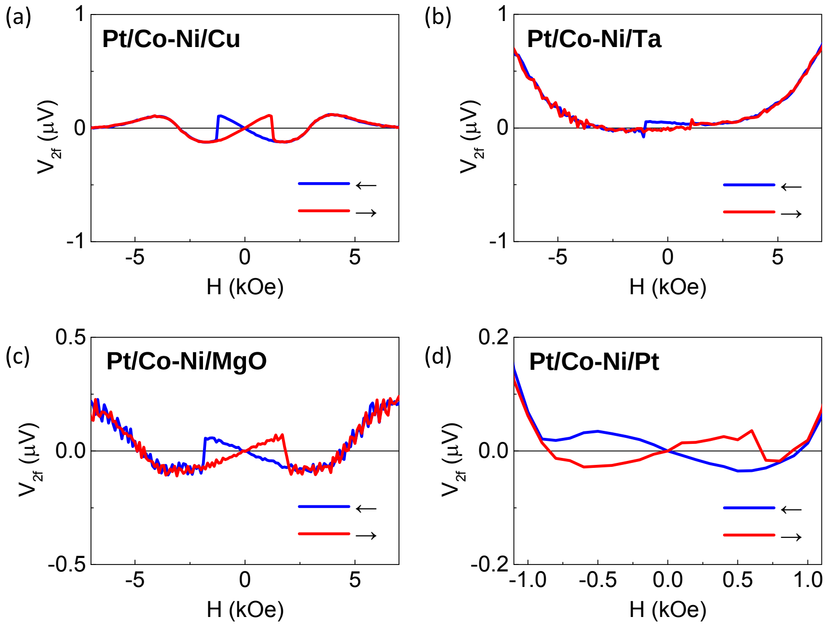

Fig. S13. Transverse second harmonic voltage (V_{2f}) in the low field region from samples with different capping layers.

The effect of PHE on the V_{2f} can be understood using the model by Qiu *et al.*[S2] The analytical expression of V_{2f} as a function of SOT effective fields ($\Delta H_{L/T}$) is obtained as follows.

$$V_{2f}^{//} = \lambda_{AHE}^{//} \frac{V_{AHE}\Delta H_L}{H_K} - \lambda_{PHE}^{//} \frac{V_{PHE}\Delta H_T}{H_K}, \tag{10}$$

$$V_{2f}^{\perp} = \lambda_{AHE}^{\perp} \frac{V_{AHE}\Delta H_T}{H_K} - \lambda_{PHE}^{\perp} \frac{V_{PHE}\Delta H_L}{H_K}, \tag{11}$$

Eq. (10) and (11) give the expressions in the longitudinal and transverse geometry, respectively, where H_K is the anisotropy field, and V_{AHE} and V_{PHE} describe the second harmonic signal from AHE and PHE, respectively. λ_{AHE} and λ_{PHE} are parameters which give the relative contributions from AHE and PHE. Both λ_{AHE} and λ_{PHE} are a function of the tilting angle of the magnetization, such that

$$
\begin{aligned}
\lambda_{AHE}^{//} &= \frac{\sin\varphi}{\sin 2\varphi \cot\left(\theta_H - \varphi\right) + 2\cos 2\varphi} \\
\lambda_{PHE}^{//} &= \frac{\sin\left(\theta_H - \varphi\right)\sin\varphi}{\cos\theta_H \sin\varphi + \sin\left(\theta_H - \varphi\right)} \\
\lambda_{AHE}^{\perp} &= \frac{1}{2\cot\left(\theta_H - \varphi\right) + 4\cot 2\varphi} \\
\lambda_{PHE}^{\perp} &= \frac{\sin\left(\theta_H - \varphi\right)\sin\varphi}{\sin\theta_H \cos\varphi}
\end{aligned}
\tag{12}
$$

where $\varphi = 90 - \theta$ is the angle between the magnetization direction and the z-axis, and θ is defined in Fig. S2(a). θ_H is the angle between the applied current and the field direction. In our measurement, θ_H is fixed as 86°.

As can be seen from Eq. (11), $V_{2f}^{\perp}$ will become negative if the following relationship holds true.

$$
\lambda_{AHE}^{\perp} \cdot V_{AHE} \Delta H_T < \lambda_{PHE}^{\perp} \cdot V_{PHE} \Delta H_L , \tag{13}
$$

We thus plot $\frac{\lambda_{PHE}^{\perp} \cdot R_{PHE}}{\lambda_{AHE}^{\perp} \cdot R_{AHE}}$ as a function of φ as shown in Fig. S14. $\frac{R_{PHE}}{R_{AHE}} = 0.557$ in Pt/Co-Ni/Cu structure is used for the calculation. The detailed measurements and results of R_{AHE} and R_{PHE} from all the samples can be found in supplementary section 2. As can be seen, $\frac{\lambda_{PHE}^{\perp} \cdot R_{PHE}}{\lambda_{AHE}^{\perp} \cdot R_{AHE}} > 1$ when φ is small. In other words, $V_{2f}^{\perp}$ becomes negative in the small field region. It should be noted that the simulated curve will be shifted up or down depending on the relative strength of H_L and H_T.

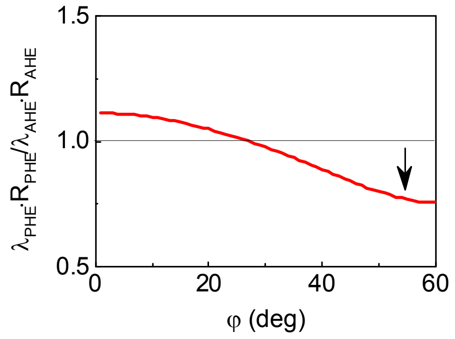

Fig. S14. Ratio of V_{2f} contributions from PHE and AHE as a function of magnetization tilting angle (φ).

As it is discussed in Supplementary section 2, PHE in our Co/Ni system is much larger than other values in HM/CoFeB systems. For example, Qiu *et al.*[S8] reported the R_{PHE}/R_{AHE} ratio to be ~0.1 for 1 nm CoFeB. Such a negligible PHE will not cause the abnormal negative V_{2f} signal.

As can be seen in Fig. S1(e), SOT fitting in the low field region is less satisfactory. This mainly attributes to the angular dependence of SOT effective fields and a large PHE. The SOT effective fields are known to have complex angular (dependence on the magnetization direction) features.[S2-4] We have calculated the angular dependence of SOT by using the model that we discussed above. The results are shown in Fig. S15.

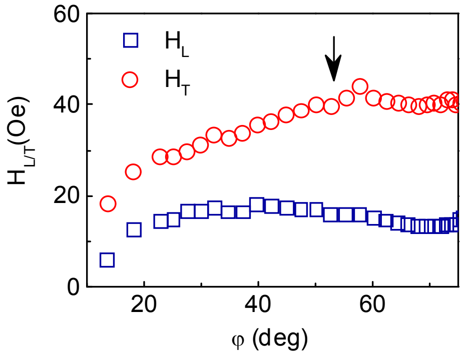

Fig. S15. SOT effective fields ($H_{L/T}$) as a function of magnetization tilting angle (φ).

For a quantitative comparison between different samples, we fit the SOT effective fields using the peak/dip values of 2nd harmonic signal, where φ is ~ 60° (marked as the black arrow in Fig. S15). As we mentioned above, PHE describes the amplitude of the correlation between H_L and H_T. the large PHE value in the device could lead to the sign change of $V_{2f}^{//}$ in the low field region. As we use the H_T value at the peak position for the fitting in Fig. S1(e), the second term of Eq. (10) is overestimated in the low field region. This leads to a negative $V_{2f}^{//}$ in the low field region as shown in Fig. S1(e). However, as shown by the open black symbol in Fig. S1(e), the measured H_T values in the low field region is not that large (see the red dots in Fig. S15). Thus we see positive $V_{2f}^{//}$ in the measured data while the fitting gives negative $V_{2f}^{//}$. For a comparison, we show the fitting results in the Pt/Co-Ni/Ta sample with zero PHE in Fig. S16. A good fitting could be obtained.

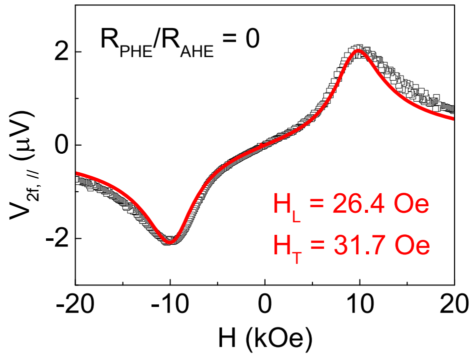

Fig. S16. The fitting of the second harmonic voltages with zero PHE. The obtained H_L and H_T values are indicated.

The complex angular dependence of H_L and H_T (on the magnetization direction) makes a direct comparison between samples difficult. Fortunately, the extracted H_L and H_T of all the samples are from $\varphi = 45 \sim 55^\circ$. Even though this method leads to the less satisfactory fitting in the field region away from the peak/dip position (especially at the low field regime), it is still capable of capturing the strength of SOT effective fields. The extracted SOT effective fields are consistent with the SOT switching current, where the sample with larger SOT fields shows a smaller SOT switching current as shown in Table 1 in the main manuscript.

11. Comparison of SOT effective fields from different fitting models

The model used in Ref. S1 and S9 fits the second harmonic voltage (V_{2f}) at low field region, therefore the magnetization is assumed to be in the z direction. In other words, the model gives SOT effective fields at $\theta \sim 90^\circ$ (θ is defined in Fig. S2a). The model in our work fits V_{2f} at a high field region ($\theta \sim 30^\circ$), therefore the signal is stronger and contains less anomalous Nernst effect (ANE), which is proportional to sin θ.[S3] To compare the difference of these two models, we have also evaluated SOTs using the model in Ref. S1 and S9. The fitting results are shown in Fig. S17.

As described in Ref. S1 and S9, SOT effective fields can be extracted using the slope of V_{2f} and the curvature of V_f. We show the V_{2f} data from the longitudinal and transverse measurement in Fig. S17 (a) and (b), respectively, from the Cu capped sample as an example. The effective fields considering PHE are then calculated by using

$$H_{L/T} = \frac{B_{L/T} \pm 2\xi B_{T/L}}{1 - 4\xi^2}, \tag{14}$$

where $B_{L/T} = -2\frac{\partial V_{2f}}{\partial H} \bigg/ \frac{\partial^2 V_f}{\partial H^2}$. ξ is the ratio of anomalous Hall resistance (R_{AHE}) and planar Hall resistance (R_{PHE}). The extracted H_{L} and H_{T} are shown as the orange symbols ("low field model") in Fig. S17 (c) and (d), respectively. For comparison, the H_{L} and H_{T} in the main manuscript, which are extracted using the model in Ref. S2, are also plotted as the blue symbols ("high field model"). As can be seen, the two models give similar SOT values, showing the Ta capped sample has enhanced SOTs, and Pt capped sample has cancelled SOTs, while MgO and Cu capped sample have intermediate SOTs. The small difference between the SOT data from the high and low field models can be understood by the angular dependence of the SOTs.[S2,3]

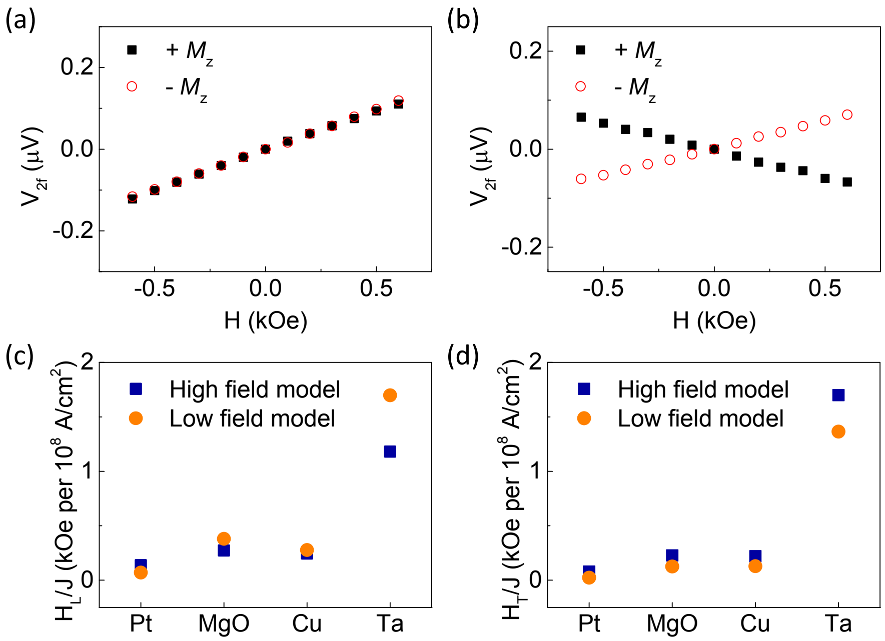

Fig. S17. (a,b) Longitudinal (a) and transverse (b) second harmonic voltage (V_{2f}) in the low field region from Cu capped sample. (c,d) Comparison of fitting results of H_{L} (c) and H_{T} (d) in the high and low field regions.

References in Supplementary Information

S1 Kim, J. *et al.* Layer thickness dependence of the current-induced effective field vector in Ta|CoFeB|MgO. *Nat. Mater.* **12**, 240-245 (2013).

S2 Qiu, X. *et al.* Angular and temperature dependence of current induced spin-orbit effective fields in Ta/CoFeB/MgO nanowires. *Sci. Rep.* **4**, 4491 (2014).

S3 Garello, K. *et al.* Symmetry and magnitude of spin-orbit torques in ferromagnetic heterostructures. *Nat. Nanotechnol.* **8**, 587-593 (2013).

S4 Lee, K.-S. *et al.* Angular dependence of spin-orbit spin-transfer torques. *Phys. Rev. B* **91**, 144401 (2015).

S5 Boulle, O. *et al.* Domain Wall Tilting in the Presence of the Dzyaloshinskii-Moriya Interaction in Out-of-Plane Magnetized Magnetic Nanotracks. *Phys. Rev. Lett.* **111**, 217203 (2013).

S6 Torrejon, J. *et al.* Interface control of the magnetic chirality in CoFeB/MgO heterostructures with heavy-metal underlayers. *Nat. Commun.* **5**, 4655 (2014).

S7 Emori, S. *et al.* Spin Hall torque magnetometry of Dzyaloshinskii domain walls. *Phys. Rev. B* **90**, 184427 (2014).

S8 Qiu, X. *et al.* Spin–orbit-torque engineering via oxygen manipulation. *Nat. Nanotechnol.* **10**, 333-338 (2015).

S9 Woo, S., Mann, M., Tan, A. J., Caretta, L. & Beach, G. S. D. Enhanced spin-orbit torques in Pt/Co/Ta heterostructures. *Appl. Phys. Lett.* **105**, 212404 (2014).
